# The Effects of Resveratrol and Apigenin on Jejunal Oxidative Injury in Ducks and on Immortalized Duck Intestinal Epithelial Cells Exposed to H_2_O_2_

**DOI:** 10.3390/antiox13050611

**Published:** 2024-05-17

**Authors:** Ning Zhou, Yongqing Cao, Youwen Luo, Lihua Wang, Ruiqing Li, Heshuang Di, Tiantian Gu, Yun Cao, Tao Zeng, Jianping Zhu, Li Chen, Dong An, Yue Ma, Wenwu Xu, Yong Tian, Lizhi Lu

**Affiliations:** 1 College of Pet Sciences, Jiangsu Agri-Animal Husbandry Vocational College, Taizhou 225300, China; 2022010534@jsahvc.edu.cn (N.Z.); 2007020097@jsahvc.edu.cn (Y.L.); wanglihua@jsnmkjzyxy6.wecom.work (L.W.); 2008010198@jsahvc.edu.cn (H.D.); 2015020298@jsahvc.edu.cn (Y.C.); 2016020323@jsahvc.edu.cn (J.Z.); 2018010374@jsahvc.edu.cn (D.A.); 2018010385@jsahvc.edu.cn (Y.M.); 2 College of Animal Science and Technology, Nanjing Agricultural University, Nanjing 210095, China; 3 State Key Laboratory for Managing Biotic and Chemical Threats to the Quality and Safety of Agro-Products, Institute of Animal Science & Veterinary, Zhejiang Academy of Agricultural Sciences, Hangzhou 310000, China; 2017805096@njau.edu.cn (Y.C.); lrqgy0601@outlook.com (R.L.); gutiantian1029@outlook.com (T.G.); zengtao@zaas.ac.cn (T.Z.); cl0010@outlook.com (L.C.); xuwenwu248@outlook.com (W.X.)

**Keywords:** resveratrol, apigenin, oxidative stress, jejunum, duck, intestinal epithelial cell

## Abstract

Oxidative stress increases the apoptosis of intestinal epithelial cells and impairs intestinal epithelial cell renewal, which further promotes intestinal barrier dysfunction and even death. Extensive evidence supports that resveratrol and apigenin have antioxidant, anti-inflammatory, and antiproliferative properties. Here, we investigated the ability of these two compounds to alleviate diquat-induced jejunal oxidative stress and morphological injury, using the duck as a model, as well as the effects of apigenin on oxidative stress induced by H_2_O_2_ in immortalized duck intestinal epithelial cells (IDECs). Ducks were randomly assigned to the following four groups, with five replicates: a control (CON) group, a diquat-challenged (DIQ) group, a resveratrol (500 mg/kg) + diquat (RES) group, and an apigenin (500 mg/kg) + diquat (API) group. We found that serum catalase (CAT) activity and total antioxidant capacity (T-AOC) markedly reduced in the RES and API groups as compared to the DIQ group (*p* < 0.05); moreover, serum S superoxide dismutase (SOD) levels increased significantly in the API group as compared to the DIQ group (*p* < 0.05). In jejunal mucosa, the malondialdehyde (MDA) content in the RES and API groups decreased more than that in the DIQ group (*p* < 0.05). In addition, the jejunal expression levels of the *NRF2* and *GCLM* genes in the RES and API groups increased notably compared with those in the DIQ group (*p* < 0.05); meanwhile, CAT activity in the RES and API groups was markedly elevated compared with that in the CON group (*p* < 0.05). In IDECs, apigenin significantly restrained the H_2_O_2_-mediated increase in MDA content and decrease in CAT levels (*p* < 0.05). Furthermore, apigenin increased the protein expression of p-NRF2, NRF2, p-AKT, and p-P38; downregulated that of cleaved caspase-3 and cleaved caspase-9; and reduced the ratio of Bax/Bcl-2 in H_2_O_2_-treated IDECs (*p* < 0.05). In conclusion, resveratrol and apigenin can be used as natural feed additives to protect against jejunal oxidative stress in ducks.

## 1. Introduction

Under large-scale and intensive feeding conditions, ducks are prone to stress reactions owing to the influence of external environmental factors, such as temperature and pesticide residues on feed. This can lead to pathological symptoms and even death, which negatively affects the interests of farmers [1]. Oxidative stress is the most common type of stress and results from an intracellular imbalance between oxidant and antioxidant systems, both of which play a crucial role in the regulation of physiological and metabolic homeostasis [2]. Furthermore, excessive generation of reactive oxygen species (ROS) results in oxidative stress, causing the disruption of redox homeostasis and ROS-mediated injury to crucial biomolecules such as DNA, proteins, and membranes [3]. ROS are normally generated during the respiratory chain reaction in mitochondria; however, environmental factors, for instance, pollution, radiation, chemicals, and drugs, can disrupt ROS production balance [4]. The level of oxidative stress can be estimated via the measurement of related parameters, such as malondialdehyde (MDA) contents, superoxide dismutase (SOD), catalase (CAT), and glutathione peroxidase (GSH-Px) activities. The concerted action of these factors serves to mitigate against the effects of oxidative stress in cells [5,6].

The mucosa of the small intestine, which is composed of epithelial cells, is in direct contact with the external environment. This renders the small intestine vulnerable to oxidative stress due to toxic food contaminants and therapeutic drugs, among other factors [7]. Meanwhile, the intestinal mucosa is the primary defense system of the host and forms a barrier against various infectious and non-infectious stimuli, thereby maintaining intestinal homeostasis [8]. Numerous studies have demonstrated that oxidative stress in the intestine can damage its morphology, disrupt its barrier function, and impair its physiological functions [9,10,11,12]. Tight junctions are key components of the intestinal barrier. Zonula occludens-1 (ZO-1), a tight junction protein, plays a major role in the maintenance of the physiological functions of tight junctions [13]. The nuclear factor erythroid 2-related factor 2 (NRF2)-Keap-ARE system plays a significant protective role against oxidative stress [14,15]. Furthermore, NRF2, a transcription factor, mitigates oxidative stress by inducing the production of antioxidants such as NAD(P)H dehydrogenase [quinone] 1 (NQO1), heme oxygenase 1 (HO-1), SOD-1, CAT, GPX-1, and glutamate cysteine ligase modifier (GCLM) [16,17].

Diquat (DIQ) is a compound that can produce a superoxide anion radical and other free radical species via cyclic reduction–oxidation processes; it is able to receive a single electron from NADPH to form a highly unstable DQ+. Then, an electron is transferred from DQ+ to molecular oxygen (O_2_) to generate O_2_^−^ with high production of SOD and H_2_O_2_ [18]. Accordingly, it is widely applied to induce intestinal oxidative stress in vivo [19,20,21,22]. Meanwhile, hydrogen peroxide (H_2_O_2_) is usually used to construct oxidative stress models in vitro [23,24,25]. In this study, diquat injection was performed to establish an oxidative stress model in ducks in vivo while H_2_O_2_ incubation was employed to generate a cellular oxidative stress model in vitro. Resveratrol is a plant polyphenolic compound belonging to the stilbene family of plant phytoalexins and is widely present in many plant-based foods such as grapes and peanuts, among others. Resveratrol has attracted substantial research attention owing to its antioxidative, anti-inflammatory, and antiapoptotic properties [26,27], and has been reported to alleviate intestinal injury caused by oxidative stress and inflammation [28,29,30,31]. Apigenin is a natural phytochemical flavonoid compound that can be found widely in several vegetables and fruits [32,33]. Studies have shown that apigenin can alleviate oxidative-stress-induced injury by activating the NRF2 pathway and enhancing the expression of antioxidative-stress-related genes [34,35]. However, few reports focus on the antioxidative effects of apigenin on duck intestine. Accordingly, the purpose of this research was to evaluate the influence of resveratrol and apigenin on the antioxidative capacity, intestinal morphology, and the expression levels of related genes in the jejunum of ducks under conditions of oxidative stress in vivo. We also explored the effects of apigenin on the viability of IDECs and cell apoptosis under oxidative stress conditions induced by H_2_O_2_.

## 2. Materials and Methods

### 2.1. Experimental Animals and Management

This experiment was conducted at the Grassland Monitoring Station, Forestry and Grassland Bureau, Yushugu District, Urumqi, Xinjiang Province. A total of 200 healthy female Sichuan Ma Ducks (average body weight of 1.05 ± 0.16 kg, 50 days old) were selected in the study. The ducks were caged in three-tier battery cages (40 × 50 × 40 cm), with 10 birds per pen. Food and water (through a nipple drinker) were offered ad libitum.

### 2.2. Experimental Design and Sample Collection

Ducks were randomly divided into the following four groups, with five replicates per group (*n* = 10 ducks per replicate): a control (CON) group, in which the ducks were fed a basal diet with sterile saline injection; a diquat-challenged (DIQ) group, in which the ducks were fed the basal diet and injected with diquat; a resveratrol + diquat (RES) group, in which the animals were fed the basal diet containing resveratrol (Huagao, Chengdu, China, 500 mg/kg) with diquat injection; and an apigenin + diquat (API) group, in which the birds were fed the basal diet containing apigenin (Shanghe, Changsha, China, 500 mg/kg) with diquat injection. The temperature and light conditions were identical across the different treatment groups. The resveratrol supplemental level was according to a previous study [36]. All birds were fed the respective diets for 10 days and then injected intraperitoneally with either 8 mg/kg body weight diquat (DIQ, RES, and API groups) in 1 mL of sterile saline to induce oxidative stress or 1 mL of sterile saline (CON group) [12]. Following NRC (1998) guidelines, the basal diets were formulated as listed in Table 1. The entire experiment lasted for 20 days, with the day of intraperitoneal injection being considered as day 0. On day 10, one duck was randomly selected from each replicate (a total of 20 ducks) for blood and jejunal tissue specimen collection. Blood samples were obtained from the axillary vein into 5 mL vacuum tubes, anti-coagulant was added, they were centrifuged at 3000× *g* for 15 min at 4 °C, and the resulting serum was stored at −20 °C until further analysis. The ducks were euthanized by intracardial administration of sodium pentobarbital (CNPGC, Beijing, China) and jugular exsanguination after overnight feed deprivation. The middle part of the jejunum was obtained and then rinsed with ice-cold phosphate-buffered saline (PBS, Solarbio, Beijing, China). A small piece of the jejunum was immediately frozen in liquid nitrogen and stored at −80 °C for the detection of antioxidant parameters and the expression of related genes. Meanwhile, 3 cm jejunal samples were fixed in 4% paraformaldehyde (Biosharp, Shanghai, China) for morphological analysis.

### 2.3. Determination of Antioxidant Parameters in Serum, Jejunum, and IDECs

Jejunal samples were immersed in PBS (1:9 *w*/*v*, g/mL), homogenized using a tissue homogenizer (Bullet Blender, Next Advance, Inc., Troy, NY, USA), centrifuged at 3500× *g* for 10 min at 4 °C, and the obtained supernatant was stored at −20 °C for detection of antioxidant parameters. MDA, SOD, GSH-Px, and CAT concentrations were detected using a fully automated biochemical analyzer (Mindray, Shenzhen, China) while the T-AOC was determined using a PerkinElmer Envision plate reader (Thermo Fisher, Waltham, MA, USA). All the relevant procedures were performed according to the manufacturers’ instructions.

### 2.4. Jejunal Histomorphology and Measurement of Jejunal Parameters

Jejunum samples were processed using hematoxylin–eosin staining methods. Three discontinuous sections of each sample were captured to measure villous height and crypt depth. Six typical fields of view were assessed for each sample using an optical microscope (Olympus BX5, Tokyo, Japan) equipped with a digital camera (Nikon, Tokyo, Japan). Image-Pro Plus 6.0 (Bethesda, Rockville, MD, USA) was used for image analysis [37].

### 2.5. Related Gene Expression Analysis

Total RNA was extracted from jejunal tissue using the E.Z.N.A. Total RNA Kit II (OMEGA Bio-Tek, GA, USA) as recommended by the manufacturer. The RNA quality was assessed using a NanoDrop (Thermo Fisher, Waltham, MA, USA) (260 nm/280 nm ratio). qPCR was conducted on a LightCycler96 (Roche, Basel, Switzerland) using SYBR Green PCR Master Mix (Vazyme, Nanjing, China) according to the manufacturers’ protocols. The cycling conditions for qPCR were 95 °C for 1 min, followed by 40 cycles of 95 °C for 10 s, 60 °C for 5 s, and 72 °C for 15 s, with an extension at 72 °C for 10 min. The 2^−ΔΔct^ method was used to calculate expression levels of target genes, with β-actin serving as an internal control. Details of the primers list in Table 2.

### 2.6. Cell Treatments

The IDECs used for cellular oxidative stress experiments were obtained from the Zhejiang Academy of Agricultural Sciences. Cell processing was performed based on our previous study [38]. Cells were pretreated with different concentrations of apigenin (0, 5, 10, 25, or 50 μM, Selleck, Houston, TX USA) for 6 h to determine the optimal treatment concentration. After pretreatment with apigenin, the cells were exposed for an additional 6 h to 400 μM H_2_O_2_ (Sigma, St. Louis, MO, USA) diluted in dimethyl sulfoxide (DMSO; Sigma, St. Louis, MO, USA). The concentrations of H_2_O_2_ were based on a previous study [38].

### 2.7. Determination of Cell Viability and Cellular ROS Levels

The viability of IDECs in each group was evaluated using a CCK-8 assay kit (Dojindo, Kumamoto, Japan) by a PerkinElmer Envision plate reader. Intracellular ROS levels were measured by a commercial ROS detection kit (Solarbio, Beijing, China) based on 2′,7′-dichlorofluorescein diacetate (DCFH-DA) staining. Both procedures were carried out in accordance with the instructions of the respective kits.

### 2.8. Flow Cytometric Analysis of the Cell Cycle and Apoptosis

Following the different treatments, cells were trypsinized, yielding cell suspensions, and then washed twice with ice-cold PBS. After incubation with RNase A solution (Sangon, Shanghai, China) at 37 °C for 30 min, the cells were incubated with propidium iodide (PI) solution (Solarbio, Beijing, China) for 30 min at room temperature, avoiding light. The rate of cell apoptosis was measured using an apoptosis detection kit (Vazyme, Nanjing, China). IDECs were resuspended in Annexin Binding Buffer and transferred to sterile flow cytometry glass tubes. Finally, after being incubated with Annexin FITC and PI at room temperature in the dark for 15 min, the cells were obtained. Flow cytometric analysis (CytoFLEX S, Beckman, Pasadena, CA, USA) was conducted per the manufacturer’s instructions. The percentage of cells in the G1 phase, S phase, and G2 phase of the cell cycle and the rate of cell apoptosis were calculated.

### 2.9. Western Blot Analysis

The treated IDEC samples were broken by homogenizer (Tissuelyser-32, Shanghai, China) after addition of RIPA buffer (Beyotime, Shanghai, China) and PMSF (Beyotime, Shanghai, China), lysed on ice for 30 min, sonicated in an ice bath for 3 min, and centrifuged at 12,000 rpm for 10 min at 4 °C. The total protein was measured using the BCA kit (Beyotime, Shanghai, China) by adding 5 × SDS loading buffer (Pujian, Wuhan, China) according to the volume of lysate and boiling at 100 °C for 5 min. Subsequently, the supernatant of IDECs was separated with sodium dodecyl sulfate polyacrylamide gel electrophoresis (SDS-PAGE, Solarbio, Beijing, China). Then, the isolated proteins were transferred onto a nitrocellulose filter membrane (Pall, BioTrace NT, Fenton, MO, USA) and the membranes were blocked with 5% non-fat milk in TBST (10 mmol/L Tris-HCl (pH 8.0), 150 mmol/L NaCl, and 0.1% Tween 20) for 1 h at room temperature. Next, the proteins were incubated with primary antibodies, including NRF2 (BS-1074, 1:500, Bioss, Beijing, China), p-NRF2 (BS2013R, 1:500, Bioss, Beijing, China), AKT (10176-2-AP, 1:1000, Proteintech, Chicago, IL, USA), p-AKT (9275S, 1:1000, CST (Danvers, MA, USA), P38 (14064-1-AP, 1:1000, Proteintech, Chicago, IL, USA), p-P38 (9211S, 1:1000, CST, Danvers, MA, USA), caspase-3 (19677-1-AP, 1:1000, Proteintech, USA), caspase-9 (10380-1-AP, 1:1000, Proteintech, USA), Bax (50599-2-Ig, 1:1000, Proteintech, Chicago, IL, USA), Bcl-2 (12789-1-AP, 1:1000, Proteintech, Chicago, IL, USA), and GAPDH (ATPA00013Rb, 1:5000, Pujian, Wuhan, China) at 4 °C overnight. After that, the proteins were incubated with secondary antibody (1:5000, Proteintech, Chicago, IL, USA) for 2 h at room temperature. Lastly, enhanced chemiluminescence (ECL, Biosharp, Chengdu, China) reagent was utilized to visualize immunoreactive bands before exposure to Tanon 4600 (Tanon, Shanghai, China). The images were analyzed by Image J software 1.42q.

### 2.10. Statistical Analysis

The statistical analysis was plotted using the GraphPad Prism 8.3 software (San Diego, CA, USA). All the data are shown as mean ± standard error (SE). Analysis of variance (one-way ANOVA) was chosen, followed by a least significant difference (LSD) post hoc test in SPSS software 27.0 (SPSS Inc., Chicago, IL, USA) when appropriate. A statistical significance was defined when *p* < 0.05.

## 3. Results

### 3.1. Serum Antioxidative Capacity

**Figure 1:**
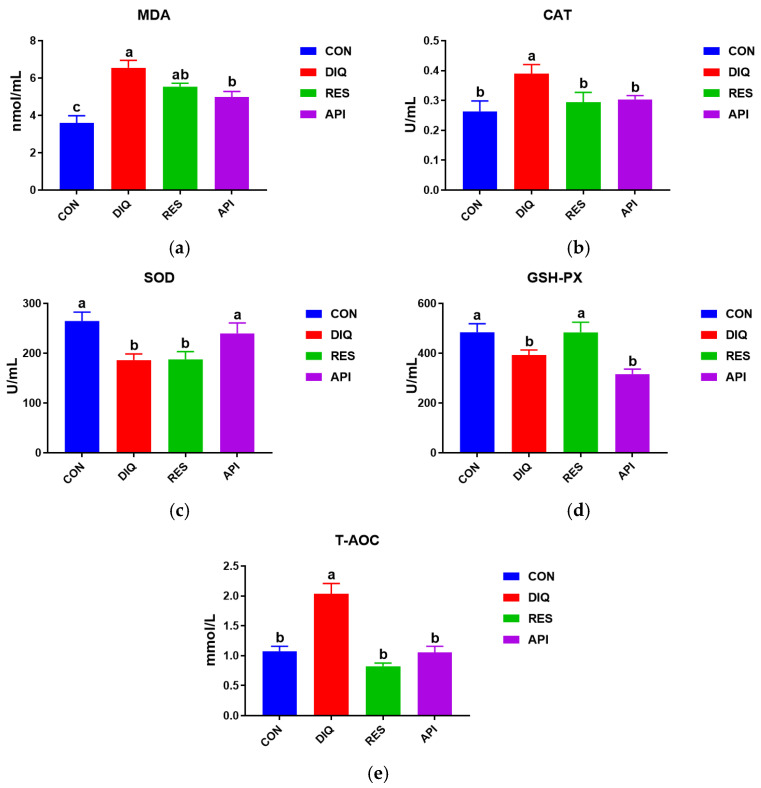
Serum antioxidative status activity of ducks fed with resveratrol and apigenin after their injection with diquat. CON: ducks fed with a basal diet and sterile saline injection; DIQ: ducks fed with a basal diet and diquat injection; RES: ducks fed with a basal diet containing 500 mg/kg resveratrol and diquat injection; API: ducks fed with a basal diet containing 500 mg/kg apigenin and diquat injection. (**a**) The MDA level of each group. (**b**) The CAT level of each group. (**c**) The SOD level of each group. (**d**) The GSH-PX level of each group. (**e**) The T-AOC level of each group. MDA, malondialdehyde; CAT, catalase; SOD, superoxide dismutase; GSH-PX, glutathione peroxidase; T-AOC, total antioxidant capacity. Analysis of variance (one-way ANOVA) was chosen, followed by a least significant difference (LSD) post hoc test. Different letters are significantly different between the groups (*p* < 0.05).

The serum antioxidative capacity of ducks was influenced by diquat challenge and dietary resveratrol and apigenin supplementation (Figure 1). As shown in Figure 1a–c, the MDA and CAT contents and the T-AOC were significantly more up-regulated in the DIQ group than in the CON group (*p* < 0.05). Meanwhile, the MDA level was lower in the REV and API groups than in the DIQ group, reaching significance with apigenin treatment (*p* < 0.05). The serum CAT concentrations and T-AOC of ducks were significantly reduced in the RES and API groups compared with those in the DIQ group (*p* < 0.05). Additionally, the SOD concentration was significantly lower in the DIQ group than in the CON and API groups (both *p* < 0.05) (Figure 1c). Furthermore, GSH-Px activity was more reduced in the DIQ group than in the CON group (*p* < 0.05); however, this inhibitory effect of diquat on GSH-Px activity was reversed with resveratrol treatment (*p* < 0.05) (Figure 1d).

### 3.2. The Redox Status of the Jejunum

**Figure 2:**
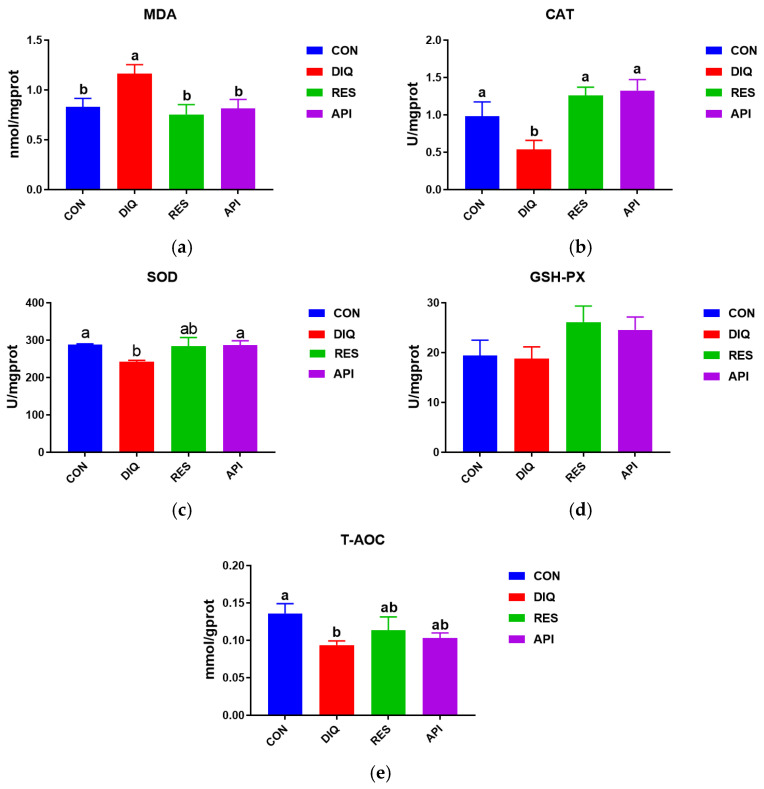
Jejunum antioxidative status activity of ducks fed with resveratrol and apigenin after their injection with diquat. CON: ducks fed with a basal diet and sterile saline injection; DIQ: ducks fed with a basal diet and diquat injection; RES: ducks fed with a basal diet containing 500 mg/kg resveratrol and diquat injection; API: ducks fed with a basal diet containing 500 mg/kg apigenin and diquat injection. (**a**) The MDA level of each group. (**b**) The CAT level of each group. (**c**) The SOD level of each group. (**d**) The GSH-PX level of each group. (**e**) The T-AOC level of each group. MDA, malondialdehyde; CAT, catalase; SOD, superoxide dismutase; GSH-PX, glutathione peroxidase; T-AOC, total antioxidant capacity. Analysis of variance (one-way ANOVA) was chosen, followed by a least significant difference (LSD) post hoc test. Different letters are significantly different between the groups (*p* < 0.05).

The levels of MDA, CAT, SOD, and GSH-Px and the T-AOC in jejunal tissue homogenates of the different groups are depicted in Figure 2. The jejunal MDA levels were markedly higher in the diquat treatment group than in the CON group (*p* < 0.05). However, dietary supplementation with resveratrol and apigenin blocked this diquat-treatment-induced decrease in the jejunal MDA level (*p* < 0.05) (Figure 2a). Moreover, CAT activity was markedly lower in the DIQ group than in the CON group (*p* < 0.05) and in the RES and API groups (both *p* < 0.05) (Figure 2b). SOD levels and the T-AOC showed a similar tendency (Figure 2c,d).

### 3.3. Jejunal Histomorphology

**Figure 3:**
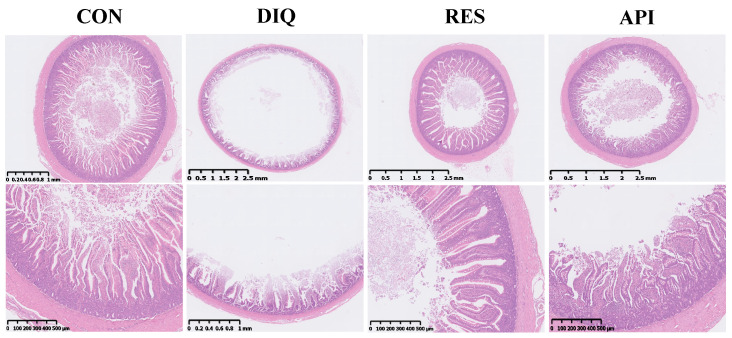
The jejunal morphological structure of ducks. CON: ducks fed with a basal diet and sterile saline injection; DIQ: ducks fed with a basal diet and diquat injection; RES: ducks fed with a basal diet containing 500 mg/kg resveratrol and diquat injection; API: ducks fed with a basal diet containing 500 mg/kg apigenin and diquat injection.

The changes in villus height, crypt depth, and the villus height/crypt depth ratio in response to diquat challenge and dietary resveratrol and apigenin supplementation in ducks are showed in Figure 3 and Table 3. In the CON group, the intestinal structure was clear, the epithelium of the mucosal layer was intact, and the intestinal villi were neatly arranged. In contrast, a large number of intestinal villi in the DIQ group appeared broken and detached, and the height of the villi and the depth of the crypts were significantly reduced in comparison to those of the CON group (*p* < 0.05). In the RES and API groups, meanwhile, the intestinal villi were more neatly arranged and the villus height was obviously greater than that of the DIQ group (*p* < 0.05). A similar trend was recorded for the crypt depth. There was no difference in the ratio of villus height/crypt depth among the four treatment groups (*p* > 0.05).

### 3.4. The Detection of Genes in the NRF2 Signaling Pathway and Encoding Antioxidant Enzymes in the Jejunum

**Figure 4:**
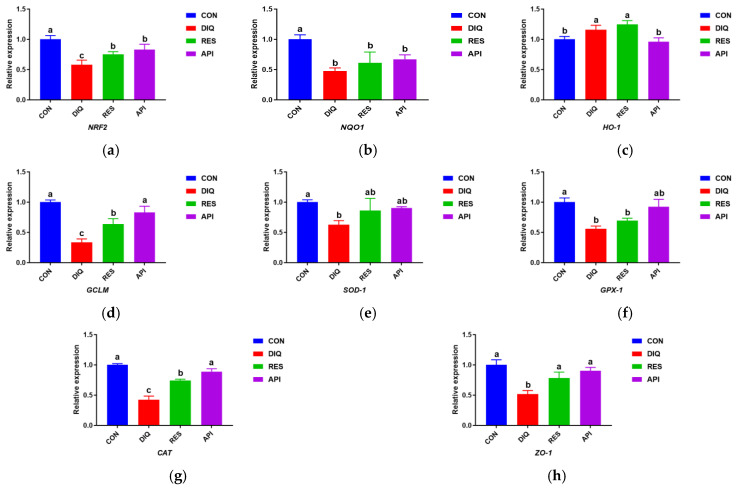
Effects of dietary resveratrol and apigenin supplementation on mRNA expression of antioxidant genes in jejunum of ducks. CON: ducks fed with a basal diet and sterile saline injection; DIQ: ducks fed with a basal diet and diquat injection; RES: ducks fed with a basal diet containing 500 mg/kg resveratrol and diquat injection; API: ducks fed with a basal diet containing 500 mg/kg apigenin and diquat injection. (**a**) The expression level of NRF2 in each group. (**b**) The expression level of NQO1 in each group. (**c**) The expression level of HO-1 in each group. (**d**) The expression level of GCLM in each group. (**e**) The expression level of SOD-1 in each group. (**f**) The expression level of GPX-1 in each group. (**g**) The expression level of CAT in each group. (**h**) The expression level of ZO-1 in each group. NRF2, nuclear factor erythroid 2-related factor 2; NQO1, NAD (P) H dehydrogenase quinone 1; HO-1, heme oxygenase-1; GCLM, glutamate cysteine ligase modifier; SOD-1, superoxide dismutase 1; CAT, catalase; GPX-1, glutathione peroxidase 1; ZO-1, zonula occludens-1. Analysis of variance (one-way ANOVA) was chosen, followed by a least significant difference (LSD) post hoc test. Different letters are significantly different between the groups (*p* < 0.05).

The results of the qPCR analysis (Figure 4a,b,d) revealed that the mRNA levels of *NRF2*, *NQO1*, and *GCLM*, genes that function in the NRF2 signaling pathway, were lower in the DIQ than in the CON group (*p* < 0.05). Additionally, the *NRF2* and *GCLM* genes’ expression levels were significantly higher in the jejunal tissue of ducks in the RES and API groups than in that of animals in the DIQ group (*p* < 0.05). No differences in the mRNA levels of *NQO1* were detected among the four groups (*p* > 0.05) (Figure 4b). Meanwhile, the *HO-1* expression level in the DIQ and RES group was markedly increased in comparison to that in the CON group (*p* < 0.05) (Figure 4c). As presented in Figure 4e–g, the jejunal expression levels of *SOD1*, *GPX1*, and *CAT* were lower in ducks of the DIQ group than in those of the CON group (*p* < 0.05). Moreover, *CAT* gene expression levels were significantly elevated in the RES and API groups (*p* < 0.05). Furthermore, the mRNA levels of *SOD1* and *GPX1* in the RES and API groups tended to be elevated compared with those in the DIQ group. Similarly, the gene expression levels of *ZO-1* were significantly upregulated in the RES and API groups compared with those in the DIQ group (*p* < 0.05) (Figure 4h).

### 3.5. The Viability of IDECs

**Figure 5:**
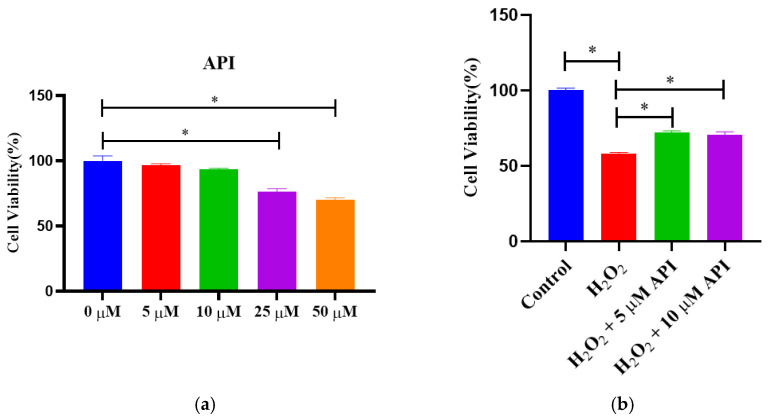
The viability of IDECs measured by CCK-8 assays. (**a**) The viability of IDEC cells with different concentrations of apigenin incubated for 6 h. (**b**) The viability of IDECs that were co-incubated with apigenin (5 μM and 10 μM) and with H_2_O_2_ (400 μM). Values for all measurements are expressed as the mean ± SE (n = 3). * *p* < 0.05 was considered statistically significant.

The viability of IDECs following treatment with different doses (0, 5, 10, 25, and 50 μM) of apigenin was detected by CCK-8 assay. At the concentrations of 5 and 10 μM, apigenin did not affect cell viability, whereas significant reductions in cell viability were observed at the concentrations of 25 and 50 μM (*p* < 0.05) (Figure 5a). In this study, we further constructed a H_2_O_2_-induced cellular model of oxidative stress. As determined in our previous work (Zhou et al., 2022), cells were exposed to 400 μM H_2_O_2_ for 6 h. We found that both the 5 and the 10 μM apigenin concentrations were effective at increasing the viability of H_2_O_2_-treated IDECs (*p* < 0.05) (Figure 5b). Based on these results, apigenin was used at the 5 μM concentration in subsequent experiments.

### 3.6. In Vitro Redox Markers 

**Figure 6:**
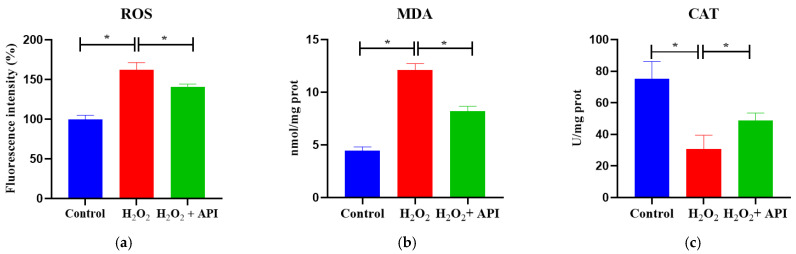
Effects of apigenin on in vitro redox markers. (**a**) The detection of ROS production in IDECs. (**b**) The detection of MDA contents in IDECs. (**c**) The detection of CAT contents in IDECs. The ROS production was determined by 2′,7′-dichlorofluorescein diacetate (DCFH-DA). The activities of MDA and CAT in IDECs were measured by commercial kits. ROS, reactive oxygen species; MDA, malondialdehyde; CAT, catalase. Control: DMSO; H_2_O_2_: 400 μM H_2_O_2_ incubated for 6 h; H_2_O_2_ + API: 400 μM H_2_O_2_ + 5 μM apigenin co-incubated for 6 h. Values for all measurements are expressed as the mean ± SE (n = 3). * *p* < 0.05 was considered statistically significant.

The ROS levels of IDECs were significantly increased under H_2_O_2_ treatment compared with the control condition (*p* < 0.05); however, this effect of H_2_O_2_ was significantly mitigated by the addition of API (5 μM) (*p* < 0.05) (Figure 6a). Moreover, 5 μM apigenin significantly inhibited the H_2_O_2_-mediated increase in MDA levels and decrease in CAT levels (*p* < 0.05) (Figure 6b,c).

### 3.7. The Effect of Apigenin on the Cell Cycle and Apoptosis in IDECs

**Figure 7:**
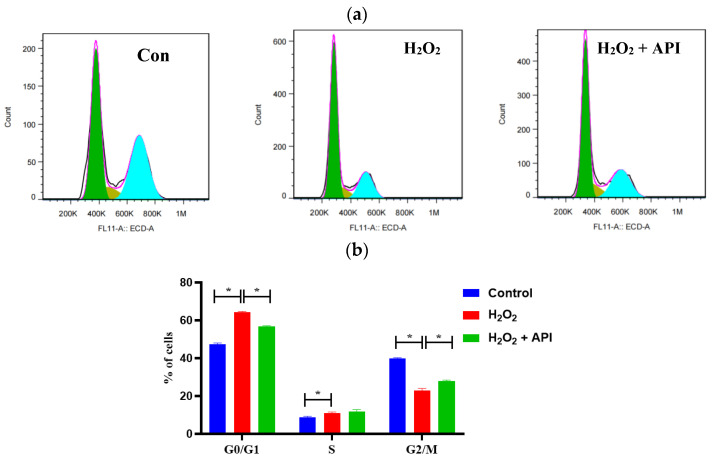
The cell cycle distribution of IDECs under oxidative stress incubated with apigenin. (**a**) Cell cycle distribution was analyzed using flow cytometry. (**b**) The data were analyzed through one-way ANOVA and are expressed as the mean ± SE (n = 3). Control: DMSO; H_2_O_2_: 400 μM H_2_O_2_ incubated for 6 h; H_2_O_2_ + API: 400 μM H_2_O_2_ + 5 μM apigenin co-incubated for 6 h. * *p* < 0.05 was considered statistically significant.

The effects of the different treatments on the IDEC cell cycle are shown in Figure 7a,b. The results of the flow cytometric analysis indicated that, following exposure to H_2_O_2_, the percentage of IDECs in the G0/G1 phase of the cell cycle was markedly up-regulated, whereas that of cells in the G2/M phase was obviously reduced (*p* < 0.05). However, co-treatment with apigenin significantly mitigated these H_2_O_2_ effects (*p* < 0.05).

**Figure 8:**
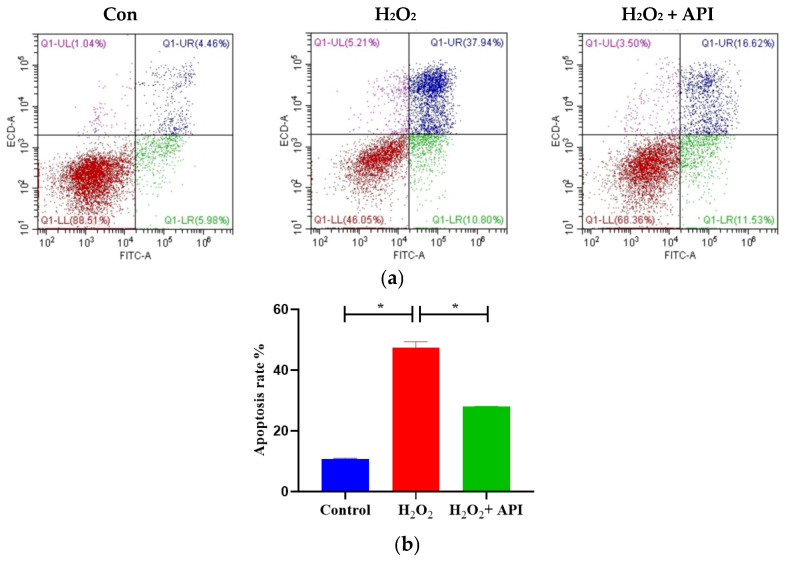
The total apoptosis rate of IDECs under oxidative stress incubated with apigenin. (**a**) The total apoptosis rates were analyzed by flow cytometry. (**b**) The data were analyzed through one-way ANOVA and values for all measurements are expressed as the mean ± SE (n = 3). Control: DMSO; H_2_O_2_: 400 μM H_2_O_2_ incubated for 6 h; H_2_O_2_ + API: 400 μM H_2_O_2_ + 5 μM apigenin co-incubated for 6 h. * *p* < 0.05 was considered statistically significant.

The apoptosis rate of IDECs in the different treatment groups was detected by flow cytometry. As depicted in Figure 8, the total apoptosis rate in IDECs was markedly upregulated under H_2_O_2_ treatment, but this effect was alleviated following the addition of apigenin (*p* < 0.05) (Figure 8a,b).

### 3.8. The Expression Levels of Antioxidant Proteins

**Figure 9:**
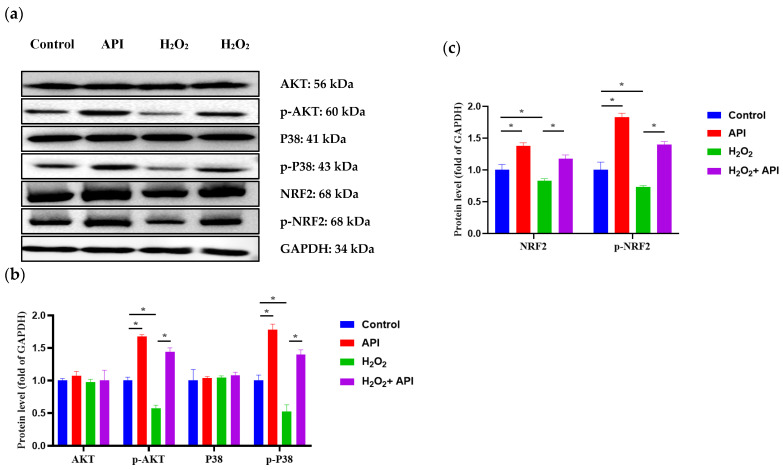
The related protein expression levels of IDECs under oxidative stress incubated with apigenin. (**a**) Protein levels of AKT, p-AKT, P38, p-P38, NRF2, p-NRF2, and GAPDH were detected by Western blot. (**b**) The levels of AKT, p-AKT, P38, and p-P38 were quantified by densitometry, and data were normalized to GAPDH. (**c**) The levels of NRF2 and p-NRF2 were quantified, and data were normalized to GAPDH. Control: DMSO; API: 5 μM apigenin incubated for 6 h; H_2_O_2_: 400 μM H_2_O_2_ incubated for 6 h; H_2_O_2_ + API: 400 μM H_2_O_2_ + 5 μM apigenin co-incubated for 6 h. The data were analyzed through one-way ANOVA and values for all measurements are expressed as the mean ± SE (n = 3). * *p* < 0.05 was considered statistically significant.

AKT and P38, as critical regulatory proteins in major cellular signaling cascades, play a key role in mediating cell survival, while the NRF2 pathway participates in the regulation of cellular oxidative stress. Accordingly, we next measured the protein levels of AKT, p-AKT, P38, p-P38, NRF2, p-NRF2, and GAPDH. No differences in AKT and P38 protein expression levels were observed between the H_2_O_2_ and CON groups. In contrast, exposure to H_2_O_2_ caused significant reduction in AKT and P38 phosphorylation levels (*p* < 0.05); however, the addition of apigenin reversed this H_2_O_2_-mediated effect (*p* < 0.05) (Figure 9a,b). Meanwhile, NRF2 and p-NRF2 protein levels were significantly lower in the H_2_O_2_ group than in the CON group (*p* < 0.05), an effect that was blocked by treatment with apigenin (*p* < 0.05) (Figure 9c).

### 3.9. The Expression Levels of Apoptosis-Related Proteins 

**Figure 10:**
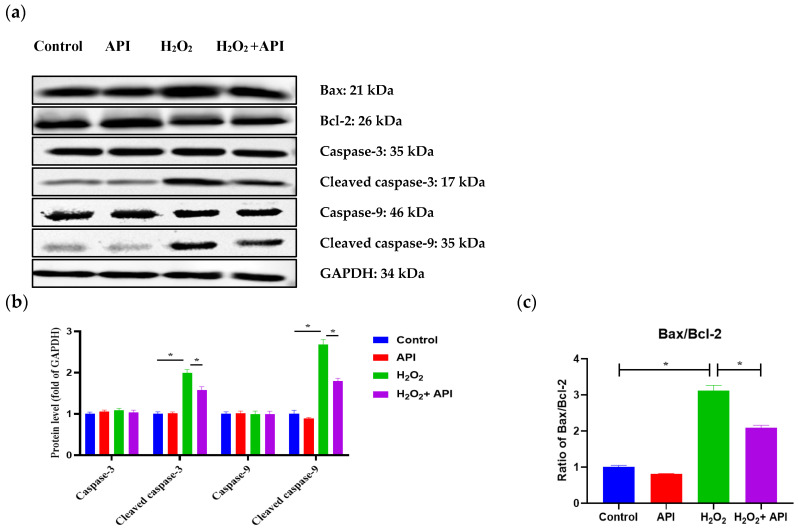
The apoptosis-related protein expression levels of IDECs under oxidative stress incubated with apigenin. (**a**) Protein levels of Bax, Bcl-2, caspase-3, cleaved caspase-3, caspase-9, and cleaved caspase-9 were detected by Western blot. (**b**) The levels of caspase-3, cleaved caspase-3, caspase-9, and cleaved caspase-9 were quantified by densitometry, and data were normalized to GAPDH. (**c**) The levels of Bax and Bcl-2 were quantified, and data were normalized to GAPDH. Control: DMSO; API: 5 μM apigenin incubated for 6 h; H_2_O_2_: 400 μM H_2_O_2_ incubated for 6 h; H_2_O_2_ + API: 400 μM H_2_O_2_ + 5 μM apigenin co-incubated for 6 h. The data were analyzed through one-way ANOVA and values for all measurements are expressed as the mean ± SE (n = 3). * *p* < 0.05 was considered statistically significant.

To determine whether the increase in the rate of cell apoptosis observed following H_2_O_2_ treatment was due to changes in the levels of apoptosis-related proteins, we next evaluated the protein expression levels of the cell-apoptosis-associated proteins, such as Bax, Bcl-2, caspase-3, cleaved caspase-3, caspase-9, and cleaved caspase-9 in IDECs by Western blot. Treatment with apigenin alone did not result in changes in caspase-3, cleaved caspase-3, caspase-9, or cleaved caspase-9 protein levels compared with that seen under the control condition (*p* > 0.05) (Figure 10a,b); however, the protein levels of cleaved caspase-3 and cleaved caspase-9 were markedly up-regulated in the H_2_O_2_ treatment group when compared with the CON group (*p* < 0.05) (Figure 10a,b). Nonetheless, apigenin supplementation significantly reduced the protein levels of cleaved caspase-3 and cleaved caspase-9 in IDECs subjected to oxidative stress (*p* < 0.05) (Figure 10a,b). The Bax/Bcl-2 ratio was more significantly increased in H_2_O_2_-induced IDECs than in control cells (*p* < 0.05), but this effect of H_2_O_2_ was markedly suppressed following the addition of apigenin (*p* < 0.05) (Figure 10a,c).

## 4. Discussion

Oxidative stress compromises epithelial integrity, increases the apoptosis of intestinal epithelial cells, and impairs intestinal epithelial cell renewal, which further results in fluid loss, electrolyte imbalance, intestinal barrier dysfunction, and even death [39,40]. Diquat, a bipyridine, redox-cycling herbicide, is one of the most commonly used compounds for constructing oxidative stress models at low concentrations. After entering the body, diquat generates free radicals and ROS in cells through redox cycling, which ultimately leads to oxidative-stress-induced damage [41]. It has also been shown that the intestine is the main target organ for diquat [42]. Therefore, in this study, diquat treatment was used to establish an oxidative stress injury model in ducks. Under oxidative stress, characterized by excessive ROS production, animals activate a complex antioxidant defense system to protect themselves against oxidative-stress-induced injury [43]. Moreover, excessive ROS can damage important cellular structures, including DNA, proteins, and membranes [44]. The concerted activity of antioxidant proteins, such as SOD, CAT, GSH-Px, and GCLM, serves to mitigate the effects of oxidative stress in cells. Accordingly, the levels of these proteins, as well as those of the products of oxidative-stress-induced damage, including MDA, can be used to quantify oxidative stress levels [3,45]. In the present study, diquat injection increased the T-AOC, MDA levels, and CAT activity while decreasing SOD and GSH-Px contents in serum; additionally, diquat treatment led to an increase in MDA contents, while a reduction in CAT and SOD activities and T-AOC in jejunal mucosa, indicating that diquat suppressed the antioxidant capacity of the jejunum of ducks, resulted in oxidative injury. Interestingly, the effects of diquat on antioxidant indices differed between serum and jejunal mucosa. In piglets, diquat treatment decreased intestinal mucosal GSH and GSH-Px levels and the T-AOC while concomitantly increasing MDA concentrations [46]. In poultry, meanwhile, Wu et al. found that MDA levels in the livers of broilers were significantly increased following diquat injection, whereas SOD and GSH-Px activities as well as the T-AOC were significantly reduced [47]. Similarly, diquat challenge was reported to downregulate GSH levels and CAT activity while increasing the concentration of MDA in the livers of broiler chickens [48]. However, relatively few studies have investigated the effects of diquat in ducks. In this work, we investigated diquat-induced oxidative stress damage in the jejunum of ducks and reached conclusions consistent with those of previous studies.

Resveratrol is a polyphenolic compound exist in a variety of plant-based foods such as nuts and grapes and is known to possess antioxidant, anti-inflammatory, and antiproliferative properties [49]. In poultry, resveratrol supplementation reportedly enhanced the activities of liver antioxidant enzymes, such as GPX and SOD, while decreasing MDA levels in heat-stressed broilers [50]. Similarly, Zhu et al. [51] reported that resveratrol supplementation significantly increased hepatic SOD activity and T-AOC in broilers. Regarding other species, meanwhile, dietary resveratrol supplementation was found to improve antioxidant capacity in weaned piglets, characterized by an increase in SOD activity in plasma [52]. Overall, our results were consistent with those of these studies, with differences in some indicators likely being due to environmental factors. Apigenin is a naturally occurring flavonoid widely found in fruits and vegetables and is documented to exert antioxidant and anticarcinogenic effects. Furthermore, given its low bioavailability, apigenin could potentially increase its health-promoting properties [53]. Studies investigating the antioxidant effect of apigenin in vivo in poultry are relatively scarce. We have previously shown that dietary supplementation with apigenin led to a notable increase in GSH-Px and SOD levels and the T-AOC and a decrease in MDA contents in pullets following diquat injection [12], which is in accordance with the results of the present study. In addition, resveratrol and apigenin supplementation were reported to enhance the antioxidant capacity of the intestinal mucosa by regulating the T-AOC and the levels of MDA, GSH-Px, CAT, and SOD, thereby relieving intestinal oxidative stress injury.

The small intestine is the main organ of absorption and digestion in ducks. It is also the first line of defense against the external environment and its morphology is related to the absorption of different substances. In the present study, we found that diquat injection disrupted the intestinal morphology of ducks, leading to the shedding of large numbers of intestinal villi and an enlarged intestinal lumen. Importantly, diquat significantly decreased the jejunal villus height and crypt depth, which is in agreement with previous studies [10,20,54]. Other works have shown that dietary resveratrol ameliorated diquat-induced intestinal morphology impairment in piglets, as evidenced by a marked increase in jejunal villi height [55]. The present study is the first to explore the ameliorative effect of apigenin supplementation on changes in intestinal morphology induced by diquat in ducks. We found that apigenin reduced diquat-induced oxidative stress and relieved the associated jejunal injury in the birds. These results provide support for further investigation of the effects of resveratrol and apigenin on the intestinal morphology of poultry.

The NRF2/HO-1 signaling pathway participates in the regulation of various stress environments by regulating antioxidant and phase 2 detoxifying enzymes, in addition to related proteins [56,57]. Under oxidative stress, NRF2 activates downstream proteins such as NQO1, HO-1, and GCLM, which enhances the coupling reaction and the expression of related antioxidant enzymes (e.g., SOD, GSH-Px, and CAT), thus protecting against oxidative-stress-induced damage [58,59]. According to our results, the jejunal abundance of *NRF2*, *GCLM*, and *CAT* mRNA was increased in the RES group compared with that in the DIQ group. Similarly, following diquat injection, apigenin supplementation led to an increase in the expression levels of *NRF2*, *GCLM*, *CAT*, and *GPX1* genes in the jejunum of ducks. Resveratrol was reported to modulate the activity of antioxidant enzymes to alleviate oxidative stress damage through the NRF2 signaling pathway in the livers of ducks, resulting from a lipopolysaccharide-induced inflammatory response [60]. One study showed that apigenin decreased irradiation-induced oxidative stress in mice by increasing NRF2 protein expression [61], while another demonstrated that apigenin promoted the nuclear translocation of NRF2 and enhanced the protein expression level of NRF2, in addition to the expression of the NRF2 target gene *NQO-1* [62]. The ZO family of proteins are key components of tight junctions. The levels of ZO-1, the most representative protein within the ZO family, serve as a marker of tight junction integrity in the epithelial barrier, and they can reflect the permeability of the intestinal epithelium [63]. Exposure to diquat has been reported to reduce ZO-1 expression at both the mRNA and protein levels [64,65], in line with our results. Studies have shown that the mRNA and protein levels of ZO-1 were increased after resveratrol treatment in mice with cyclophosphamide-induced immunosuppression [66]. Previous results demonstrated that apigenin was found to restore the integrity of the intestinal barrier by increasing the expression of ZO-1 [67,68]. Collectively, our results indicated that the NFR2 signaling pathway is involved in the resveratrol- and apigenin-mediated alleviation of diquat-induced oxidative stress, and that resveratrol and apigenin protect intestinal permeability by enhancing ZO-1 expression under oxidative stress.

Given that duck epithelial cell lines are not commercialized and cannot be purchased directly, immortalized duck small intestinal epithelial cells must be generated [38]. In this study, IDECs were used as a tool to explore the effect of apigenin on oxidative stress in the duck intestine. H_2_O_2_ is relatively stable and can penetrate cytomembranes and induce cellular damage through oxidation; accordingly, it is commonly used to construct cellular oxidative stress models [69,70]. Here, we used exposure to H_2_O_2_ to establish a model of oxidative-stress-induced damage in IDECs. We hypothesized that the protective effect of apigenin was mediated through the attenuation of oxidative damage and the improvement of jejunal epithelial cellular antioxidant activity. We found that at the 5 and 10 μM concentrations, apigenin was effective at increasing cell viability under H_2_O_2_ treatment. Furthermore, the addition of apigenin at the 5-μM concentration greatly reduced ROS and MDA levels, while significantly increasing CAT activities, thereby relieving H_2_O_2_-induced oxidative stress injury in IDECs. Similar to our results, Jittapalapong et al. [71] found that apigenin addition significantly increased ROS and MDA contents in TM4 cells under oxidative stress. Additionally, apigenin reportedly decreased ROS and MDA levels and increased CAT activity in PC12 cells [72].

We further investigated the effect of apigenin on the cell cycle in IDECs. The addition of apigenin significantly increased the percentage of IDECs in the G2/M phase relative to that observed under H_2_O_2_ treatment, which agrees with previous studies [73,74,75]. The PI3K/AKT and P38 MAPK signaling pathways are involved in NRF2-dependent transcription in diverse cell types as well as in apoptosis in response to oxidative-stress-induced injury [76,77,78,79]. Studies have shown that apigenin can induce breast cancer cell apoptosis through the PI3K/AKT/NRF2 pathway, thereby inhibiting the growth of breast cancer [80]. Moreover, apigenin treatment induced extrinsic apoptosis and autophagic cell death through the PI3K/AKT pathway in human gastric cancer cells [81]. In the present study, we observed that apigenin upregulated the protein levels of NRF2, p-NRF2, p-AKT, and p-P38 in cells under H_2_O_2_ treatment, indicating that apigenin attenuates oxidative stress injury by activating relevant signaling pathways.

Bax and Bcl-2, which belong to the Bcl-2 protein family, play an important role in the regulation of cell apoptosis, while caspases participate in regulation of apoptotic cell death [82,83,84]. Previous investigators have reported that apigenin is able to attenuate the hepatic apoptosis by decreasing the expression of Bax and caspase-3 and increasing the expression of Bcl-2 in rats [85]. Our results showed that apigenin treatment led to a notable downregulation of the levels of cleaved caspase-3 and cleaved caspase-9 in IDECs under oxidative stress; meanwhile, apigenin treatment mitigated the increase in the Bax to Bcl-2 ratio induced by oxidative stress in IDECs. These findings suggested that apigenin alleviates cell apoptosis by modulating Bax, Bcl-2, cleaved caspase-3, and cleaved caspase-9 protein expression.

## 5. Conclusions

Collectively, our findings suggest that resveratrol and apigenin are able to attenuate acute jejunal injury induced by diquat injection and reduce jejunal morphological changes in ducks via their antioxidant activity. Our results further indicate that resveratrol and apigenin can modulate the mRNA levels of genes in the NRF2 pathway, thereby protecting against oxidative-stress-induced injury in the jejunum of ducks. In IDECs, our data suggest that apigenin is able to alleviate oxidative stress via the PI3K/AKT and P38 MAPK signaling pathways, and restrain apoptosis by regulating the protein levels of cleaved caspase-3, cleaved caspase-9, Bax, and Bcl-2. Thus, resveratrol and apigenin can be exploited as potential natural antioxidants applied in the relief of intestinal oxidative stress damage, which contributes to the development of the farming industry.

## Figures and Tables

**Table 1:** Ingredients and nutrient composition of the basal diet.

Ingredients	Content (%)	Nutrient Levels	Content
Corn	53.2	Metabolic energy ^2^ (MJ/kg)	12.20
Soybean meal	26.8	Crude protein, %	17.85
Wheat bran	10.6	Lysine, %	1.1
Soybean oil	3.61	Cysteine + methionine, %	0.68
Limeston	1.77	Calcium, %	3.50
NaCl	0.3	Available phosphorus, %	0.37
DL-methionine	0.22		
CaHPO ^3^	1.5		
Vitamin-mineral premix ^1^	2.00		
Total	100		

^1^ The premix provided the following nutrients per kilogram of diet: VA, 11,500 IU; VD3, 33,000 IU; VE, 25 IU; VK3, 33 mg; VB1, 3 mg; VB3, 150 mg; VB6, 4.5 mg; VB12, 0.03 mg; niacin, 0.2 mg; pantothenic acid, 12 mg; folic acid, 0.8 mg; biotin, 0.15 mg; choline chloride, 100 mg; Fe, 80 mg; Cu, 100 mg; Mn, 50 mg; Zn, 90 mg; Se, 0.2 mg; I, 0.4 mg. ^2^ Calculated by NRC (1998) nutrient requirement for ducks. ^3^ Analyzed content.

**Table 2:** Primer sequence.

Genes	Primer (from 5′ to 3′)	Product Size (bp)	Accession Number
*NRF2*	F: GTTGAATCATCTGCCTGTGG	153	NM_001310777.1
	R: TAAGCTAGGTGGTCGAGTGC		
*NQO1*	F: AAGAACCCCGAGCACTTCGT	142	XM_027466610.1
	R: CCTCTCCCATCTCCGTCTCGT		
*GCLM*	F: CAGTCATTATTGCCCCGCCTC	131	XM_027462629.1
	R: CCATTCGTGTGCTTTGACGTT		
*HO-1*	F: TTCCCAGAAACACGGCTCT	145	KU048806
	R: TTCCCTCCAGTTTCTGCCGTA		
*SOD-1*	F: CCTCGGCAACGTGACTGCTA	159	XM_027449207.2
	R: ACTTGGCTATTCCGATGACACC		
*GPX-1*	F: CAGTACATCATCTGGTCGCC	185	XM_027467953.2
	R: CCTGGATCTTGATGGTTTCG		
*CAT*	F: CTTTACAATGCCATAGCCCAT	168	XM_027458335
	R: CCTCCGCAAAGTAATTGACAGG		
*ZO-1*	F: ACGCTGGTGAAATCAAGGAAGAA	179	XM_038184904
	R: AGGGACATTCAACAGCGTGGC		
*β-actin*	F: ATGTCGCCCTGGATTTCG	135	EF667345.1
	R: CACAGGACTCCATACCCAAGAAT		

F, forward primer; R, reverse primer; *NRF2*, nuclear factor erythroid 2-related factor 2; *NQO1*, NAD (P) H dehydrogenase quinone 1; *GCLM*, glutamate cysteine ligase modifier; *HO-1*, heme oxygenase-1; *SOD-1*, superoxide dismutase 1; *CAT*, catalase; *GPX-1*, glutathione peroxidase 1; *ZO1*, zonula occludens-1.

**Table 3:** Effects of resveratrol and apigenin on jejunal morphological indicators in ducks.

Items	CON	DIQ	RES	API
Villus height (μm)	578.33 ^a^ ± 29.13	362.71 ^c^ ± 19.94	563.33 ^a^ ± 19.19	469.71 ^b^ ± 30.19
Crypt depth (μm)	173.17 ^a^ ± 5.29	92.07 ^c^ ± 8.72	151.67 ^a^ ± 10.08	143.43 ^b^ ± 5.39
Villus height/crypt depth	3.35 ± 0.18	4.18 ± 0.53	3.83 ± 0.37	3.29 ± 0.23

Note: ^a,b,c^ Means without a common superscript with a row differ significantly (*p* < 0.05). Analysis of variance (one-way ANOVA) was chosen, followed by a least significant difference (LSD) post hoc test. CON: ducks fed with a basal diet and sterile saline injection; DIQ: ducks fed with a basal diet and diquat injection; RES: ducks fed with a basal diet containing resveratrol and diquat injection; API: ducks fed with a basal diet containing apigenin and diquat injection.

## Data Availability

Data are contained within the article.
